# Effect of Micro- and Nanomagnetite on Printing Toner Properties

**DOI:** 10.1155/2014/706367

**Published:** 2014-01-19

**Authors:** Maryam Ataeefard, Ebrahim Ghasemi, Mona Ebadi

**Affiliations:** ^1^Department of Printing Science and Technology, Institute for Color Science and Technology, P.O. Box 16765-654, 1668814811 Tehran, Iran; ^2^Department of Inorganic Pigment and Glazes, Institute for Color Science and Technology, 1668814811 Tehran, Iran; ^3^Department of Chemistry, Islamic Azad University, East Tehran Campus, Tehran, Iran

## Abstract

Toner is a main component of electrophotographic printing and copying processes. One of the most important ingredients of toner is magnetite (Fe_3_O_4_) which provides the tribocharging property for toner particles. In this study, nano- and microparticles of Fe_3_O_4_ were synthesized using the coprecipitation method and different amounts of lauric acid as a surfactant. The synthesized nano and micro Fe_3_O_4_ was then used as the charge control agent to produce toner by emulsion aggregation. The Fe_3_O_4_ and toner were characterized by X-ray powder diffraction (XRD), atomic gradient force magnetometry (AGFM), dynamic laser scattering (DLS), particle size analysis, differential scanning calorimetry (DSC), and scanning electron microscopy (SEM). The results show that the optimum amount of surfactant not only reduced particle size but also reduced the magnetite properties of Fe_3_O_4_. It was found that the magnetite behavior of the toner is not similar to the Fe_3_O_4_ used to produce it. Although small-sized Fe_3_O_4_ created toner with a smaller size, toners made with micro Fe_3_O_4_ showed better magnetite properties than toner made with nano Fe_3_O_4_.

## 1. Introduction

Toner is a composite powder that contains polymer, pigment, magnetite, and additives which is used for electrophotography (EP) printing. In the last two decades, EP has become a legitimate alternative to analog print production technologies [1].

The application of electrophotography varies from manufacturer to manufacturer, but the basic principle is the same. Initially, a photoconductive belt or roller is uniformly charged. Next, the image area is selectively discharged, usually by a laser. Subsequently, toner is brought into contact with the photoreceptor. Upon contact with the photoreceptor, toner particles attach to the discharged image areas of the photoreceptor. This toner image is then transferred to the substrate, where it is bonded using heat and pressure. Finally, the photoreceptor is cleaned of residual charges and toner in preparation for the next image (see Figure S1 in the Supplementary Material available online at http://dx.doi.org/10.1155/2014/706367) [2].

As previously mentioned, toner is manufactured by combining polymer, pigment, magnetite, and additives. The resulting mass is extruded and mechanically ground to produce toner particles small enough for use in electrophotography [3]. Mechanically ground toner has limitations; one of the most important limitations is minimum particle size. Mechanical milling limits the minimum particle size to about 7 *μ*m. In addition to limiting particle size, mechanically milled toners exhibit a wide distribution of particle sizes and shapes that adversely affect image quality [4].

One solution to this is the use of chemically prepared toner (CPT). Unlike mechanical milling, CPT toner is synthesized from nanometer-sized particles using one of several chemical processes, such as suspension polymerization [5], chemical milling [6], or emulsion aggregation (EA) [7]. CPT toners have smaller particle sizes and more uniform mean particle size distributions. EA is chemical process that grows very small, uniformly sized toner particles from even smaller (submicron) components [1]. The EA process can deliver the desired narrow particle size distribution required for excellent color image quality [8].

Technically, the printing mechanism in electrophotography requires the toner to be magnetic [9]. Iron oxide is the strongest natural magnetic mineral; it occurs in many forms [10]. Specifically, magnetite (Fe_3_O_4_) has scientific and industrial applications [11, 12] because of its magnetic properties and its color, density, and hardness [13]. Magnetite is used for pigments, building materials, and, especially, in printing toner to control the magnitude of the toner charge [10, 14].

Nanosized materials have many modern applications, although macrosized materials also have advantages, like ease of production [15]. Magnetite nanoparticles have noteworthy characteristics such as superparamagnetism, highfield irreversibility, high saturation field, and extra anisotropy or shifted loops after field cooling [16, 17]. From a practical point of view, the method of preparation of the nanomaterial represents one of the greatest challenges at present. There are currently several methods to prepare magnetic Fe_3_O_4_ particles, such as microemulsions [18], laser-pyrolysis [19], sonochemical synthesis [20], and chemical coprecipitation [21].

The one-step coprecipitation process is a predominant method of producing nano- and micromagnetite because only two chemicals are needed and production costs are lower. Few researches has focused on the controlled synthesis [22, 23] and effect of Fe_3_O_4_ properties, for special applications like electrophotography [14] and no research has focused on a comparison of applications for nano and micro Fe_3_O_4_ produced via coprecipitation method.

The aim of this study is to control the size of the Fe_3_O_4_ particles, from nano- to microsized, using one-step coprecipitation method with different amounts of surfactant and compares the effect of size on the properties of printing toners produced via EA methods.

## 2. Material and Preparation

### 2.1. Materials

Ferrous chloride (FeCl_2_·4H_2_O), ferric chloride (FeCl_3_·6H_2_O), ammonia, and lauric acid were purchased from Merck and used without further purification. The polymer used in this study was a styrene-acrylic resin (NS88; Simab Resin Co., Tehran). A polyethylene emulsion wax (EE 95, Kala Kar Co., Tehran) and a carbon black pigment (Printex U, Degussa-Evonik, Germany) were also used in the experiments. Polyaluminum chloride was used as a coagulation agent.

### 2.2. Preparation

#### 2.2.1. Preparation of the Magnetite

The coprecipitation method was used to synthesize Fe_3_O_4_ nanoparticles. The process for the Fe_3_O_4_ nucleation from a salt solution occurs in the reaction [17]:

(1) FeCl2·4H2O+2FeCl3·6H2O+8NH4O ⟶Fe3O4+20H2O+8NH4Cl

A 0.5 M solution of FeCl_3_·6H_2_O and FeCl_2_·4H_2_O was prepared at a molar ratio of 2 : 1 and stored in a glass reactor. A 25 mL of ammonia aqueous solution (25%) was then charged in solution by stirring with a mechanical stirrer; adding the ammonia aqueous solution to the mixture brought the pH value to 11. Lauric acid was added and intensely stirred at 60°C for 30 min. The precipitate was washed several times with deionized water. During the whole process, N_2_ gas was purged from the reactor. All precipitates were collected by centrifugation and dried at room temperature to remove water content.  Table 1 shows the experimental sets and Fe_3_O_4_ particle types and sizes.

#### 2.2.2. Preparation of the Printing Toner

All toners in this study were prepared using the following procedure [5, 7]. 24.5 g styrene-acrylic latex, 2 g carbon black, 3 g wax, 5 g Fe_3_O_4_ nanoparticles, and 120 g deionized water were mixed manually at room temperature for 15 min in a glass beaker and then mixed using a homogenizer (with 5000 RPM agitation speed) for 5 min. This mixture was then continuously mixed for 60 min at room temperature (with 2000 RPM agitation speed). Afterward, a solution of 0.6 g coagulation agent in acid was added dropwise over 10 min until the pH of the mixture was adjusted to 2.

A gel was formed during this process and the viscosity of the suspension changed dramatically from an initially Newtonian, water-like fluid to a very shear thinning, paste-like gel. The temperature of the mixture was then raised to 50°C within 30 min, while the gel was mixed and was held at this temperature for another 60 min. Then, the temperature of the mixture was raised to 96°C within 30 min and held at this temperature for further 60 min. The mixture was neutralized using a sodium hydroxide solution after raising the temperature. Finally, the mixture was cooled to 25°C, after which the produced microparticles were isolated from the liquid, washed to remove divalent ions, filtered, and dried (see Figure S2). The Fe_3_O_4_ particles synthesized as described above were used to explore the effect of Fe_3_O_4_ particle size on toner properties.

### 2.3. Characterization

#### 2.3.1. Characterization of the Magnetite Particles and Printing Toner

The dried powder samples of magnetite nanoparticles were characterized using an X-ray powder diffractometer (XRD) with Cu-K*α* radiation at a wavelength of 1.54 Å (Eindhoven, The Netherlands). The particle size distribution and morphology of the synthesized particles were investigated using a Philips FE/CM200 Transmission Electron Microscopy (TEM, Eindhoven, The Netherlands) and a dynamic laser scattering (Malvern Zen 3600, UK). The saturation magnetization was measured using an alternative gradient force magnetometer (AGFM-150).

The size and size distribution of the toner particles were determined using a Particle Size Analyzer (PSA, Mastersizer2000, Malvern, UK). Evaluation of the particle size distribution was done using the span parameter:

(2) $$\begin{array}{c} \mathrm{span}=\frac{\left(D90-D10\right)}{D50}, \end{array}$$

where *D*50 represents the diameter (*μ*m) at which half of the population lies below this value. Similarly, 90 percent of the distribution lies below the *D*90, and 10 percent of the population lies below the *D*10 [20].

A melting point meter (Buchi, Switzerland) and a differential scanning calorimeter (Pyris 6, Perkin Elmer, Germany) were employed to investigate the thermal behavior of the toner. Scanning Electron Microscopy (SEM, KYKY-EM3200, China) was utilized to investigate the shape and morphology of the toner particles. The saturation magnetization was measured using the above-mentioned AGFM at room temperature.

## 3. Results and Discussion

### 3.1. Magnetite Properties

**Figure 1:**
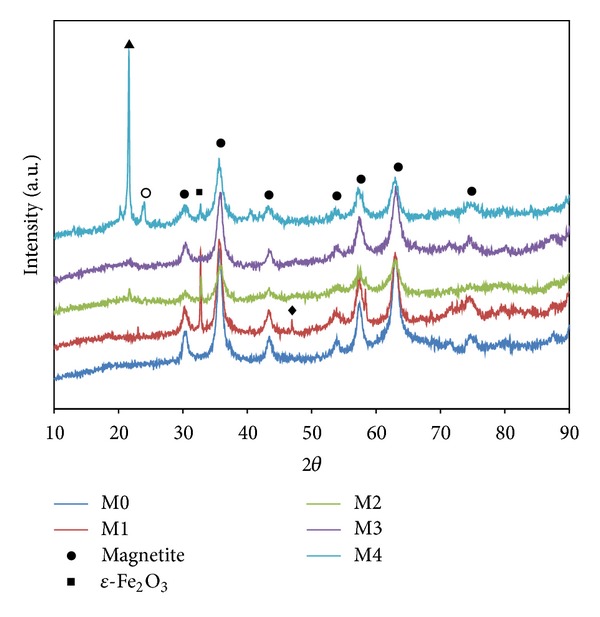
The XRD pattern of the synthesize Fe_3_O_4_ particles with various amounts of surfactant.

The XRD pattern of the precipitate is shown in Figure 1. It is clear that the magnetite was the main phase in all synthesized samples, which corresponds to JCPDS card 00-011-0614. The relatively broad peaks indicate the ultrafine nature and small crystallite size of the particles. Some samples (M1, M2, and M4) showed an intermediate phase for *ε*-Fe_2_O_3_; all the peaks matched JCPDS card number 1309-1.

This phase formation may be related to the aggregation oxygen adsorption and the conditions of production, such as pH and concentration surfactant [22]. Excessive amounts of surfactant lead to aggregation, indicating side phase formation. XRD results show that, in M0 with no surfactant and M3 with optimum surfactant, no *ε*-Fe_2_O_3_ formation was seen. In samples M1, M2, and M3, this side phase can be seen.

**Figure 2:**
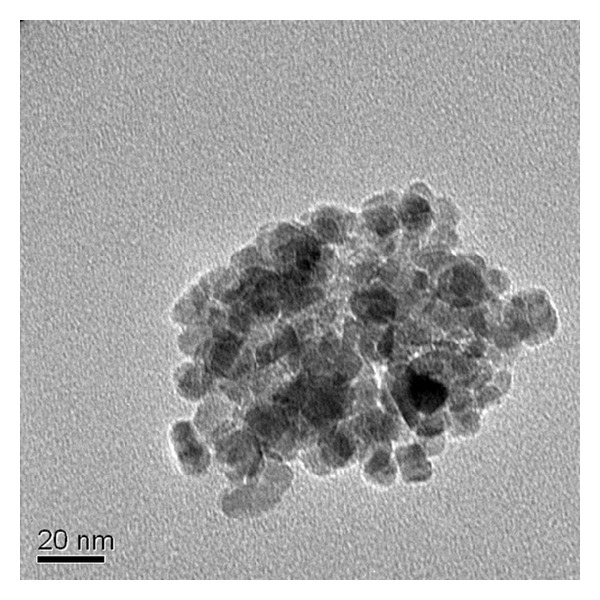
TEM images of Fe_3_O_4_ particles (M3).

TEM result for M3 sample can be seen in Figure 2. It can be seen that the particles are in the range of 6–20 nm, with a mean diameter of 11 nm. The average crystallite size was found using the Sherrer equation [13]:

(3) $$\begin{array}{c} D=\frac{0.9\lambda }{\left(B\cos\theta \right)}, \end{array}$$

where *D* is the average core diameter of the particles, *λ* is the wavelength of the incident X-ray, and *B* is the full width in radians subtended by the half-maximum intensity width of powder peak *θ*.

**Figure 3:**
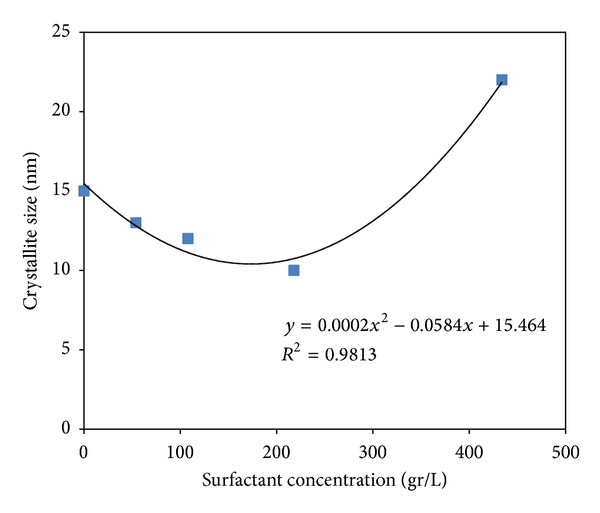
The average crystal size of synthesize Fe_3_O_4_ particles with various amounts of surfactant by Sherrer's equation.

**Figure 4:**
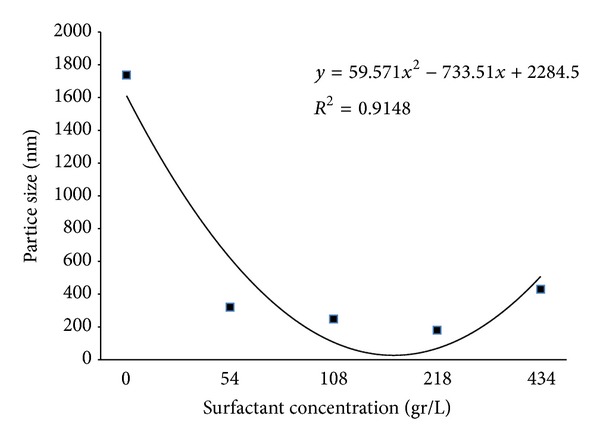
The average particle size of synthesize Fe_3_O_4_ particles with various amounts of surfactant.

Figure 3 shows the crystallite size for all samples. It can be seen that increasing the amount of surfactant to an appropriate concentration (M3) first decreased particle size and then increased it. The appropriate amount of surfactant acts as a barrier, reducing the particle size. Excessive amounts of surfactant lead to aggregation and the formation of large particles.  Figure 4 shows the hydrodynamic size of Fe_3_O_4_ particles versus surfactant concentration. It is clear that the surfactant is very effective in reducing size. The increase in size in the M4 sample may be related to micelle formation [22].

**Figure 5:**
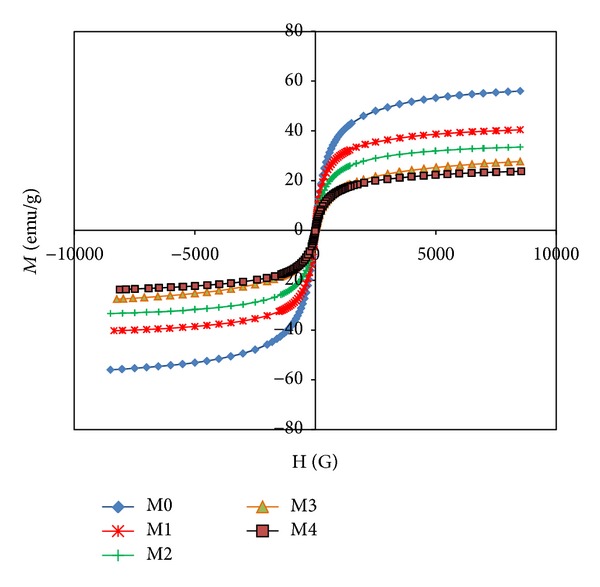
Magnetite properties of synthesize Fe_3_O_4_ particles with various amounts of surfactant.

**Figure 6:**
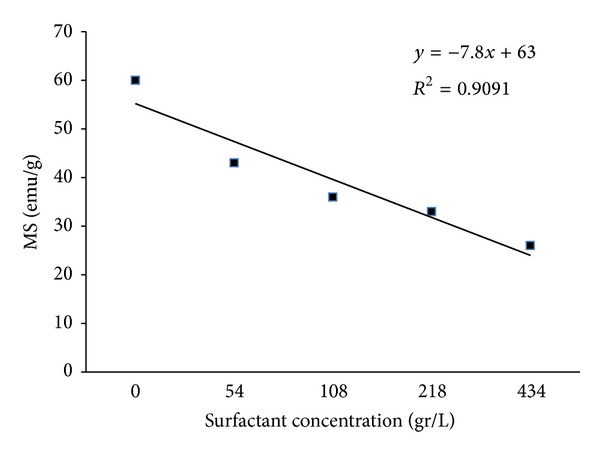
Magnetite saturation of synthesize Fe_3_O_4_ particles with various amounts of surfactant.

Figure 5 shows the hysteresis loop of the assynthesized powder that exhibits a low remanent magnetization value similar to superparamagnetic behavior.  Figure 6 shows that increasing the amount of surfactant decreased the variation in saturation magnetization of the nanoparticles. The reduction in Fe_3_O_4_ saturation magnetization may be a result of the presence of a nonmagnetic layer of surfactant (lauric acid) on the particle surface that reduces saturation magnetization (Ms).

### 3.2. Printing Toner Properties

The effects of magnetite size change from nano to macro on toner structural properties were tested in different sets. The particle size and particle size distribution (span) of the toner particles are shown in Table 2. The particle size and particle size distribution results show that all toner particle sizes and size distributions are in the appropriate range (~7–13 nm). Nano- and smaller-sized magnetite particles produced toner with smaller particle sizes and particle size distributions, which in turn produced higher-quality images [1].

**Figure 7:**
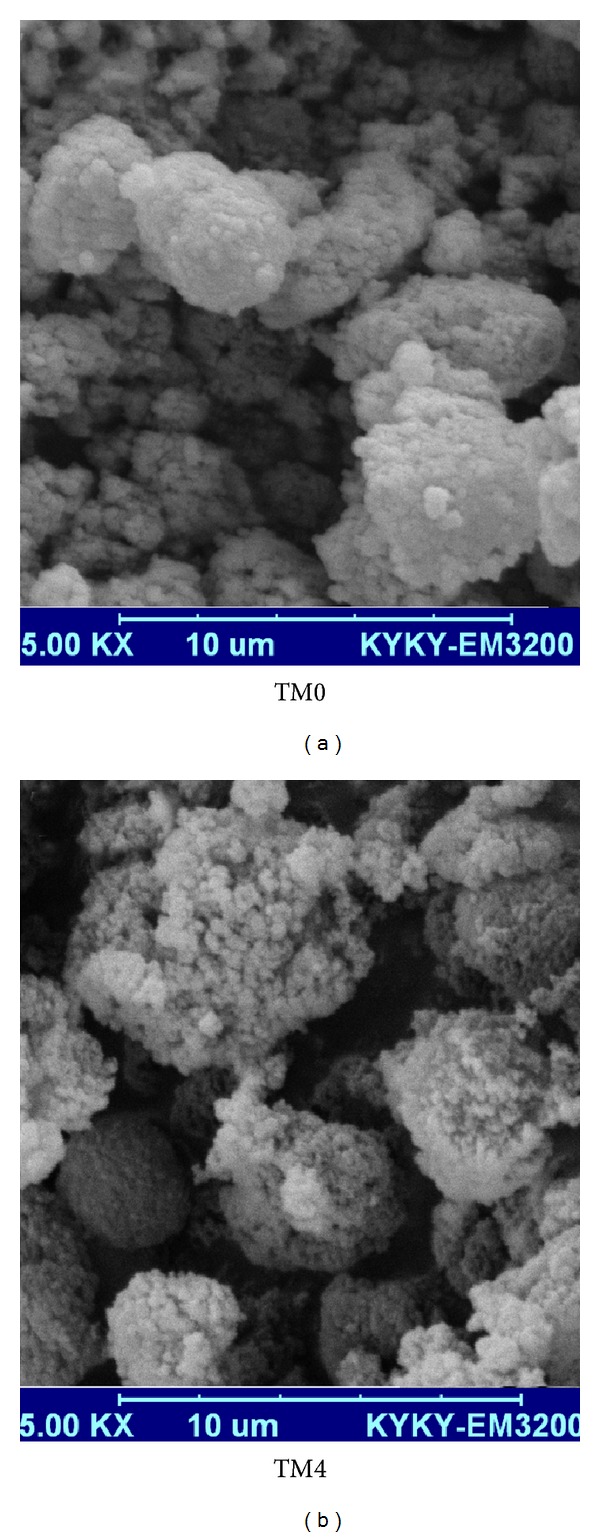
SEM images of toner synthesize with various types of Fe_3_O_4_ particles.

The particle shape of the toner depended on the aggregation temperature and the glass transition temperature (*T*_*g*_) of the polymer, which can be adjusted by controlling the temperature and other processing parameters, such as the agitation rate and using more spherical ingredients [24].  Figure 7 shows SEM micrographs of the toner particles. It can be seen that all toner particles are nearly spherical in shape and that changing the size of the magnetite did not affect the shape of the toner particles.

The thermal characteristics of the toner, especially *T*_*g*_, have a direct effect on fixing the properties of the toner on the substrate. *T*_*g*_ determines the fusing temperature of the toner. Generally, a moderate *T*_*g*_ value is required for the toner to have appropriate fixing properties. Too high *T*_*g*_ results in high energy consumption during the printing process. Conversely, if *T*_*g*_ is too low, the toner will stick to the printer cartridge. Suitable fixing properties for Original Equipment Manufacturer (OEM) toners for energy-efficient laser printing requires a *T*_*g*_ value in the range of 50°C to 70°C [25, 26].

Table 2 shows the results of differential scanning calorimetry analysis of the printing toners and the softening points of all synthesized toner samples. The appropriate amount of *T*_*g*_ and softening point may be related to the *T*_*g*_ of the polymer component (styrene-acrylic resin) used in toner formulation (*T*_*g*_ = 51.12°C). In other words, the *T*_*g*_ value and softening point of the toner sample show that the synthesized toner has the thermal characteristics of OEM toners for use in EP printing.

**Figure 8:**
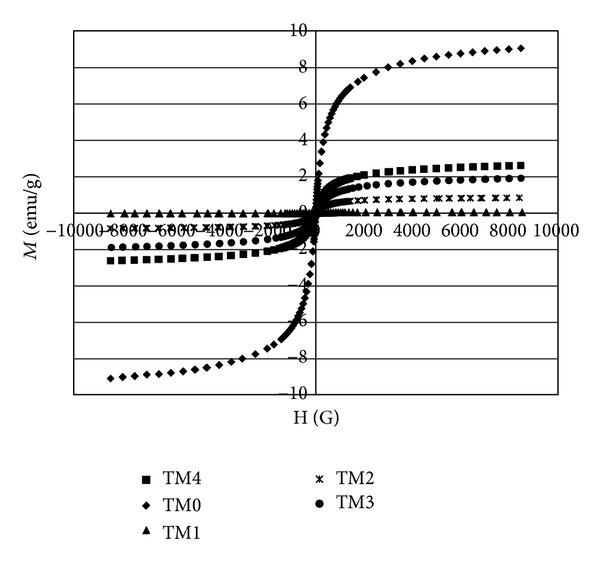
Magnetite properties of toner synthesize with various types of Fe_3_O_4_ particles.

Figure 8 shows the hysteresis loop of the printing toners. Similar to the magnetites used to prepare them, the diagram exhibits a low remanent magnetization value that indicates the presence of a semisuperparamagnetic fraction in the material [13].

**Figure 9:**
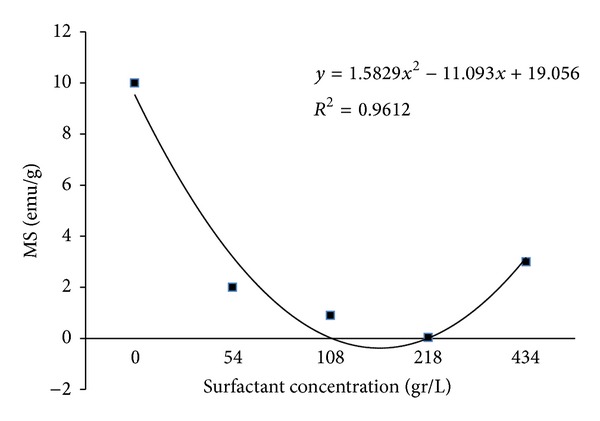
Magnetite saturation of toner synthesize with various types of Fe_3_O_4_ particles.

Figure 9 shows the variation of saturation magnetization of the toners. It was expected that, alike the magnetites used to prepare the toners, Ms decrease as the toner particles size decreased, while this behavior was only observed for TM1 and TM5 (M1 contained no surfactant and M5 contained extrasurfactant). Ms for the other toners were either much more or much less than TM5. In the other words, it was expected that Ms would vary in order of TM1 > TM2 > TM3 > TM4 > TM5, but the results showed that variation was in the order of TM1 > TM5 > TM2 > TM3 >TM4. This is similar to the variation in particle and crystal size. It seems that nanosized magnetite oxidized rapidly in acid used in toner synthesis and loses its magnetic ability. At the same time, bigger magnetite sizes are more resistant to acid, which is why that they show higher Ms. Evidently, using nanosized magnetite produces smaller toner particles but reduces the magnetic properties of the printing toner. In conclusion, the most suitable magnetite size for the optimum toner characteristics is the macrosized magnetite.

## 4. Conclusions

This study investigated the synthesis of Fe_3_O_4_ particles by coprecipitation with a focus on the effect of surfactant on the formation of Fe_3_O_4_ nano- and macroparticles. XRD revealed that the assynthesized particles were either *ε*-Fe_2_O_3_ or Fe_3_O_4_ with an average size of 15 nm to 22 nm. It was shown that changing the amount of the acid lauric as surfactant from 0 to 434 gr/lit has a major, but nonlinear, relationship to the hydrodynamic size of Fe_3_O_4_ (varies from 180 to 1737 nm). AGFM results revealed that the Fe_3_O_4_ are semisuperparamagnetic and Ms decreased as the particle size or amount of lauric acid increased, although this behavior was not observed in the electrophotographic toners synthesized with those Fe_3_O_4_ particles. The toner produced with macrosized Fe_3_O_4_ showed better magnetic properties, while image quality decreased for toners with larger particle sizes. All produced printing toners had suitable particle size (9 to 13 *μ*m) with spherical shape and appropriate thermal properties.

## Supplementary Material

Figure S1: Schematic overview of the electrophotographic process.
Figure S2: The variation of pH and temperature during the synthesis process of the toner composites.

## Figures and Tables

**Table 1:** The defined experimental sets for Fe_3_O_4_ particles synthesize with variations in lauric acid amount and defined experimental sets for toner synthesize with various types of Fe_3_O_4_ particles.

Magnetite sample	Surfactant concentration (gr/lit)	Magnetite colloidal appearance
M0	0	Unstable
M1	54	Semistable
M2	108	Stable
M3	218	Stable
M4	434	Un-stable

**Table 2:** Particle size, glass transition temperature, and softening point of synthesize toner samples.

Toner sample	Particle size (*μ*m)	Span	Glass transition temperature (°C)	Softening point (°C)
TM0	9	1.75	51.25	134
TM1	7.2	1.40	51.36	135
TM2	6.9	0.95	51.31	130
TM3	6.2	0.87	53.22	131
TM4	13	1.95	52.78	132
