# Supplementary material for: Effect of Micro- and Nanomagnetite on Printing Toner Properties
Source: ScientificWorldJournal. 2014 Jan 19;2014:706367. doi: 10.1155/2014/706367 (PMC3916107; doi:10.1155/2014/706367)

**Figure S1:** Schematic overview of the electrophotographic process.


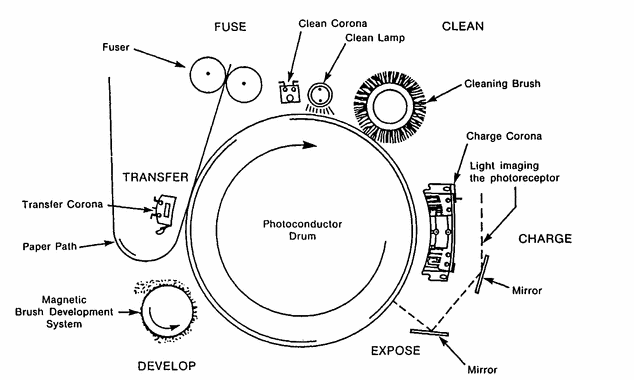


**Figure S2:** The variation of pH and temperature during the synthesis process of the toner composites.


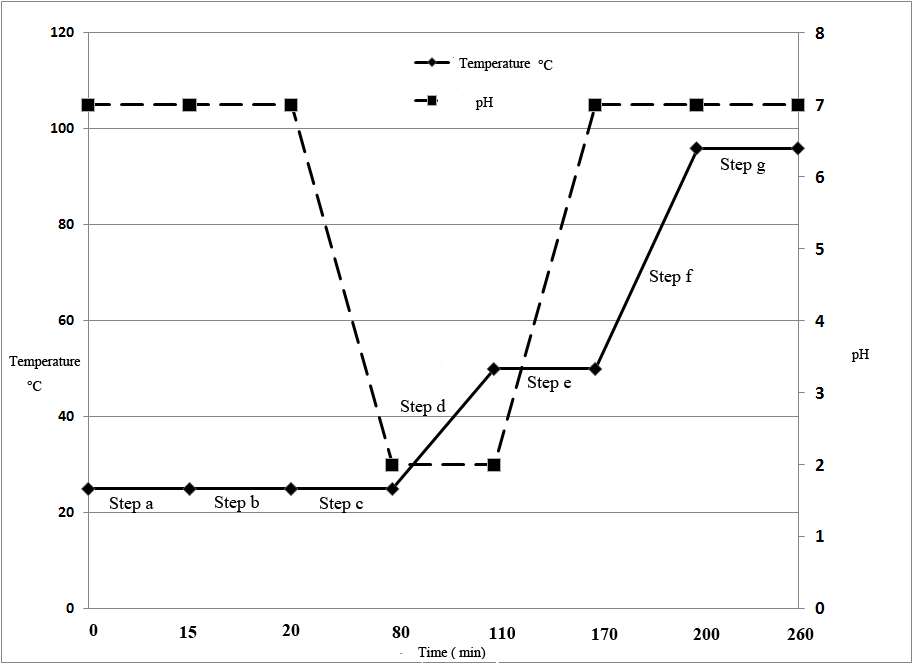

Supplement: Supplementary file 1 — Figure S1: Schematic overview of the electrophotographic process. Figure S2: The variation of pH and temperature during the synthesis process of the toner composites. [file 706367.f1.docx]
